# Smart Ocean: A New Fast Deconvolved Beamforming Algorithm for Multibeam Sonar

**DOI:** 10.3390/s18114013

**Published:** 2018-11-17

**Authors:** Jie Huang, Tian Zhou, Weidong Du, Jiajun Shen, Wanyuan Zhang

**Affiliations:** 1 Acoustic Science and Technology Laboratory, Harbin Engineering University, Harbin 150001, China; huangjie_iron_man@hrbeu.edu.cn (J.H.); zhoutian@hrbeu.edu.cn (T.Z.); shenjiajun@hrbeu.edu.cn (J.S.); zhangwanyuan@hrbeu.edu.cn (W.Z.); 2 Key Laboratory of Marine Information Acquisition and Security, Harbin Engineering University, Harbin 150001, China; 3 College of Underwater Acoustic Engineering, Harbin Engineering University, Harbin 150001, China

**Keywords:** fast deconvolved beamforming, tunnel effect, high-frequency multibeam sonar, high bearing resolution, low side lobe

## Abstract

A new fast deconvolved beamforming algorithm is proposed in this paper, and it can greatly reduce the computation complexity of the original Richardson–Lucy (R–L algorithm) deconvolution algorithm by utilizing the convolution theorem and the fast Fourier transform technique. This algorithm makes it possible for real-time high-resolution beamforming in a multibeam sonar system. This paper applies the new fast deconvolved beamforming algorithm to a high-frequency multibeam sonar system to obtain a high bearing resolution and low side lobe. In the sounding mode, it restrains the tunnel effect and makes the topographic survey more accurate. In the 2D acoustic image mode, it can obtain clear images, more details, and can better distinguish two close targets. Detailed implementation methods of the fast deconvolved beamforming are given, its computational complexity is analyzed, and its performance is evaluated with simulated and real data.

## 1. Introduction

Smart Ocean engineering systematically integrates a variety of marine sensors, including underwater acoustic sensors and underwater acoustic sensor networks [1,2,3,4], to develop and utilize a rich variety of resources. Multibeam sonar is one of the main underwater acoustic sensors for remotely sensing seabed characteristics. Multibeam sonar can provide the full sea depth, wide coverage, and high precision seabed topography, surficial seabed type information, and two-dimensional images of targets in the water column, all of which play an important role in the smart ocean, and the performance of multibeam sonar is seriously affected by the robust high-resolution beamforming algorithm.

In the field of beamforming, we usually multiply the data vector obtained at the same time by the weighted vector of a fixed value to obtain the maximum response in one or more directions, which is usually called conventional beamforming (CBF). The advantage of CBF is that it is robust and it can work in the condition of a low signal-to-noise ratio (SNR). However, CBF suffers from fat beams and high-level side lobes, and it cannot detect a weak signal among loud interfering sources.

To overcome the shortcomings of CBF, some high-resolution beamforming methods based on the inverse of the signal covariance matrix have been proposed. The minimum-variance distortionless response (MVDR) and multiple signal classification (MUSIC) are representative methods. These algorithms yield narrow beam widths and low side lobe levels, and therefore they allow for better interference rejection and detection of targets with a small bearing separation. At the same time, these algorithms are known to be sensitive to the signal mismatches [5]. To minimize the sensitivity for producing a wider beam in the target/interference look direction while keeping the side lobe level low, the MVDR algorithm has been modified with additional constraints, such as the white noise gain (WNG) constraint [6] or distributed sources constrains [7]. In addition, another problem with these high-resolution algorithms is that the signal covariance matrix is usually not known, which is replaced by the sample covariance matrix. Assuming that the signal is stationary, the sampled covariance matrix requires many samples of signals (usually 2–3 times the number of sensors) to guarantee rapid convergence and minimum power loss in the beam output [8]. For a nonstationary environment, such as a moving target with a high bearing rate, the number of samples available for a covariance matrix that is estimated without violating the stationary condition can be relatively small, which causes a snapshot deficient condition [9]. These high-resolution algorithms usually have a poor performance under these conditions.

Deconvolved beamforming was first proposed by Yang [5], and it has been applied to low-frequency line arrays, which works very well, yields narrow beam widths, and low side lobe levels. However, the Richardson–Lucy (R–L) deconvolution algorithm, which is used in Yang’s paper, requires a huge amount of calculation. Based on Yang’s paper, we further study the implementation of the R–L deconvolution algorithm and propose a new fast R–L deconvolution algorithm, which can greatly improve the efficiency of the original R–L algorithm. We applied the new fast R–L deconvolution algorithm to high frequency line arrays, and it obtained satisfactory results.

## 2. Beamforming and Deconvolution

### 2.1. Conventional Beamforming

**Figure 1:**
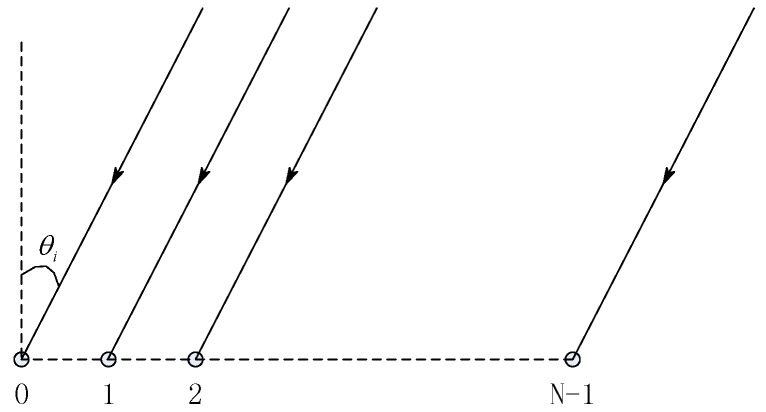
The geometric shape of the linear array.

In this section, we consider a horizontal line array (HLA) with N sensors, uniformly spaced with a separation d. The signal from a narrow band source located at the far field of the array, with a look direction $\theta_{i}$ (see Figure 1), arrives at the array as a plane wave and is denoted by $r=S_{i}a_{i}+n$. $S_{i}$ is the signal strength; $a={\left[a_{0}^{i},a_{1}^{i},\ldots ,a_{N-1}^{i}\right]}^{T}$ is the steering vector related to $\theta_{i}$, defined by $a_{n}^{i}=e^{jn\frac{2\pi }{\lambda }d\sin\theta }$ and n=0,1,…,N−1; the superscript *T* denotes vector transpose; j denotes the imaginary number; and n denotes the noise vector.

The signal amplitude $S_{i}$ is a random under the environment where the multibeam sonar works which obeys the K distribution [10,11,12]. Consequently, the beam power averaged over several snapshots of data, can be given by

(1) $$\;B\left(\sin\theta \right)=\langle {\left|W^{H}r\right|}^{2}\rangle =W^{H}\langle {\mathrm{rr}}^{H}\rangle W=\overset{M}{\underset{i=1}{\sum }}\langle \left|S_{i}^{2}\right|\rangle {\left|\frac{\sin c\left(\pi N\left(d/\lambda \right)x_{i}\right)}{\sin c\left(\pi \left(d/\lambda \right)x_{i}\right)}\right|}^{2}\;$$

where $W={\left[w_{0},w_{1},\ldots ,w_{N-1}\right]}^{T}$, $w_{n}=\frac{1}{N}e^{jn\frac{2\pi }{\lambda }d\sin\theta }$ and $\langle {\mathrm{rr}}^{H}\rangle$ is the signal sample covariance matrix (〈 〉 denotes the average over the samples). Because the signals that from different sources are uncorrelated or incoherent against each other, thus $\langle S_{i}^{H}S_{j}\rangle =\langle {\left|S_{i}\right|}^{2}\rangle \delta_{\mathrm{ij}}$.

(2) $$\;W^{H}\langle {\mathrm{rr}}^{H}\rangle W=W^{H}\left[\overset{M}{\underset{i=1}{\sum }}\overset{M}{\underset{j=1}{\sum }}a_{i}\langle S_{i}^{H}S_{j}\rangle a_{j}^{H}\right]W=\overset{M}{\underset{i=1}{\sum }}\langle {\left|S_{i}\right|}^{2}\rangle {\left|W^{H}a_{i}\right|}^{2}\;$$

### 2.2. Deconvolved Beamforming

#### 2.2.1. The Equivalent Process of CBF

We rewrite Equation (1) as follows:

(3) $$\;B\left(\sin\theta \right)=\int_{-1}^{1}B_{p}\left(\sin\theta -\sin\alpha \right)S_{p}\left(\sin\alpha \right)d\sin\alpha \;$$

where

(4) $$B_{p}\left(\sin\theta \right)={\left|\frac{\sin c\left(\pi N\left(d/\lambda \right)\sin\theta \right)}{\sin c\left(\pi \left(d/\lambda \right)\sin\theta \right)}\right|}^{2}$$

This is called the beam pattern, which relates to the array element number, the array element spacing, and the signal wavelength. That is, this function is known to be a certain horizontal linear array.

(5) $$\;S_{p}\left(\sin\theta \right)=\left[\overset{M}{\underset{i=1}{\sum }}\langle {\left|S_{i}\right|}^{2}\rangle \delta \left(\sin\theta -\sin\theta_{i}\right)\right]\;$$

Equation (5) is the source power distribution function, which consists of several impulse functions that denote targets from different directions. 

**Figure 2:**
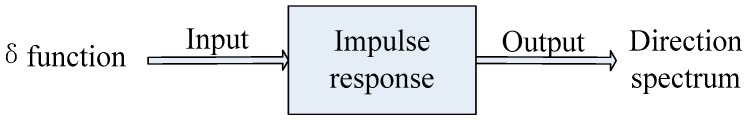
The equivalent process of CBF.

Therefore, CBF can be regarded as a process where the impulse function passes a linear system and the output is the direction spectrum. Figure 2 shows the process. Its physical explanation is that the real target becomes blurred when it is observed because of the finite length of the array, and the blurring process is the convolution of the real azimuth spectrum of the target and the beam pattern of the array. The ambiguous results we observed (i.e., those obtained by conventional beamforming) are the convolution of the true azimuth spectrum of the target and the array beam pattern. Following this idea, we can deconvolute the ambiguous results with the azimuth spectrum of the array, and then we can get the real azimuth spectrum of the target.

The deconvolution can be done with the direction spectrum B(sinθ) (the output of CBF) and the beam pattern $B_{p}\left(\sin\theta \right)$, and then the impulse function of the signal is obtained. The impulse function is only valued at the variable value 0 and valued zero at any other positions so that the very narrow main lobe can be obtained and there is no side lobe. Thus, high-resolution beamforming is achieved.

#### 2.2.2. R–L Algorithm

At present, there are many algorithms for deconvolution and the R–L algorithm is one of them. The R–L algorithm is an iterative algorithm based on Bayes conditional probability theorem [13,14]. The algorithm is only applicable to non-negative real numbers. The beam pattern can be used to deconvolve the beam power because the beam power and beam pattern are nonnegative.

Equation (3) can be rewritten to consider the noise, and the discrete model of CBF is given by:

(6) $$\;F=F_{p}⊗S+n\;$$

where ***S*** denotes source power distribution, ***F*** denotes beam power, $F_{p}$ denotes beam pattern (also known as the point scattering function), ***n*** denotes noise, and ⊗ denotes convolution operation symbol. Then, the iterative R–L algorithm [13] is given by

(7) $$\;{\hat{S}}_{k+1}={\hat{S}}_{k}\left(F_{p}*\frac{F}{F_{p}⊗{\hat{S}}_{k}}\right)\equiv \psi \left({\hat{S}}_{k}\right)\;$$

where ${\hat{S}}_{k}$ denotes the estimate of S in the *k* iteration and ∗ denotes correlation operation. ψ(…) is referred to as the R–L function. Deconvolution can be realized by utilizing the recursion expression above.

#### 2.2.3. Fast R–L Algorithm

A detailed description of the R–L recursive algorithm is referred to as Algorithm 1, from which it can be estimated that, per iteration, the multiplication number is $2N^{2}+N$ (N is the number of beams), the division number is N, and the addition number is 2N(N−1). The time consumption of a multiplication or division is far greater than that of an addition or subtraction on the computer or digital signal processor (DSP). Therefore, the computation complexity of an algorithm mainly depends on how many times there are multiplications and divisions. As shown, the computational complexity of the R–L algorithm is $O\left[N^{2}\right]$. In other words, the amount of calculation is proportional to the square of the number of the beams. As N becomes larger, the amount of calculation increases sharply.
**Algorithm 1:** R–L algorithm.Initialization: ${\hat{S}}_{0}=F$ (or ${\hat{S}}_{0}={\left[1,1,\ldots ,1\right]}_{1\times N}^{T}$), N is the number of elements of ${\hat{S}}_{0}$ or the number of beams. for k = 1, 2, 3, … {  ${\hat{F}}_{k}=F_{p}⊗{\hat{S}}_{k}$; Estimate ***F*** at the iteration time k
 ${\hat{S}}_{k+1}={\hat{S}}_{k}\left(F_{p}*\frac{F}{{\hat{F}}_{k}}\right)$; Estimate ***S*** at the iteration time k+1
}

It can be seen through the careful analysis of the R–L algorithm that the reason why it requires a huge computation is because of the convolution and correlation operations, and their computation complexity is $O\left[N^{2}\right]$. According to the convolution theorem, the spectrum of the convolution of two signals in a time domain is equal to the product of their spectrum in the frequency domain. The spectrum of the signal can be efficiently calculated utilizing the fast Fourier transform (FFT) technique, and the computation complexity of FFT is between O[N] and $O\left[N^{2}\right]$. Therefore, by utilizing the convolution theorem and FFT technique, the computation complexity of the R–L algorithm can be greatly reduced. The specific implementation methods is referred to as Algorithm 2, from which we can estimate, per iteration, that the multiplication number is $8N\log_{2}N+9N$, the division number is N, and the addition number is $8N\log_{2}N+4N$. The computation complexity is between O[N] and $O\left[N^{2}\right]$.
**Algorithm 2:** Fast R–L algorithm.Initialization: ${\hat{S}}_{0}=F$ (or ${\hat{S}}_{0}={\left[1,1,\ldots ,1\right]}_{1\times N}^{T}$), N is the number of elements of ${\hat{S}}_{0}$, or the number of beams. $H_{p}=fft\left(F_{p}\right)$; Calculate the fast Fourier transform of $F_{p}$ and fft(…) denotes the fast Fourier transform operation. for k =1, 2, 3, … { ${\hat{H}}_{{\hat{S}}_{k}}=fft\left({\hat{S}}_{k}\right)$; Calculate the Fourier transform of ${\hat{S}}_{k}$
${\hat{H}}_{{\hat{F}}_{k}}=H_{p}{\hat{H}}_{{\hat{S}}_{k}}$; Calculate the Fourier transform of ${\hat{F}}_{k}$
${\hat{F}}_{k}=ifft\left({\hat{H}}_{{\hat{F}}_{k}}\right)$; Calculate the inverse Fourier transform of ${\hat{H}}_{{\hat{F}}_{k}}$ and ifft(…) denotes inverse fast Fourier transform operation.  $H_{\mathrm{temp}}=fft\left(\frac{F}{{\hat{F}}_{k}}\right)$;  $\mathrm{temp}=ifft\left(H_{p}^{*}H_{\mathrm{temp}}\right)$; $H_{p}^{*}$ denotes the conjugate of $H_{p}$
${\hat{S}}_{k+1}={\hat{S}}_{k}\mathrm{temp}$
}.

#### 2.2.4. Accelerated R–L Algorithm and Fast Accelerated R–L Algorithm

Biggs and Andrews pointed out the problem that the speed of the iteration of the R–L algorithm is too slow in Reference [15], and they proposed a method of vector extrapolation to accelerate the speed of iteration. The technique calculates the direction as the difference between the current iteration and the previous iteration. If ${\hat{S}}_{k}$ is the iterated point, $S_{k}^{\prime }$ is the predicted point, ${\hat{S}}_{k}-{\hat{S}}_{k-1}$ is the direction vector, and $\alpha_{k}$ is the acceleration parameter, then

(8) $$\;S_{k}^{\prime }={\hat{S}}_{k}+\alpha_{k}\left({\hat{S}}_{k}-{\hat{S}}_{k-1}\right)\;$$

where

(9) $$\;{\hat{S}}_{k}={\hat{S}}_{k-1}\left(F_{p}*\frac{F}{F_{k-1}^{\prime }}\right)\;$$

(10) $$\;F_{k}^{\prime }=F_{p}⊗S_{k}^{\prime }\;$$

(11) $$\;\alpha_{k}=\frac{g_{k-1}^{T}\cdot g_{k-2}}{g_{k-2}^{T}\cdot g_{k-2}}\;$$

(12) $$\;g_{k}={\hat{S}}_{k+1}-S_{k}^{\prime }\;$$

The detailed derivation of $\alpha_{k}$ is given in the appendix of Reference [15].

The amount of calculation of a single iteration is greater than Algorithm 1 and Algorithm 2, but the total iteration, which needs to reach the same result, becomes less. A description of its iterative algorithm is given as Algorithm 3, from which we can estimate, per iteration, that the multiplication number is $2N^{2}+4N$, the division number is N+1, the addition number is $2N^{2}+N-2$, and the subtraction number is 2N. For a single iteration of Algorithm 3, we can also utilize the FFT technique to reduce the number of calculations of the convolution and correlation. Finally, we obtain the optimum algorithm (hereinafter referred to as Algorithm 4) that requires the least amount of calculation, from which we can estimate, per iteration, that the multiplication number is $8N\log_{2}N+12N$, the division number is 2N+1, the addition number is $2N^{2}+N-2$, and the subtraction number is 2N. It is worth noting that Algorithm 3 and Algorithm 4 start to iterate after k is greater than 2. When *k* equals 1 or 2, it should still use Algorithm 1 and Algorithm 2, respectively, because $\alpha_{k}$ cannot be calculated when *k* equals 1 or 2. The multiplication times of the four algorithms of a single iteration is shown in Table 1. It is proved in the next section that the total amount of calculation that is needed to reach the same result satisfies Algorithm 1 > Algorithm 2 > Algorithm 3 > Algorithm 4 when the beam number is small, and Algorithm 1 > Algorithm 3 > Algorithm 2 > Algorithm 4 when the beam number is large. Table 2 summarizes the differences between the four algorithms.
**Algorithm 3**: Accelerated R–L algorithm.Initialization: ${\hat{S}}_{0}=F$ (or ${\hat{S}}_{0}={\left[1,1,\ldots ,1\right]}_{1\times N}^{T}$), N is the number of elements of ${\hat{S}}_{0}$ or the number of beams. for k =3,4,… { $S_{k}^{\prime }={\hat{S}}_{k}+\alpha_{k}\left({\hat{S}}_{k}-{\hat{S}}_{k-1}\right)$; Predict S at the iteration time k
 $F_{k}^{\prime }=F_{p}⊗S_{k}^{\prime }$; Predict ***F*** at the iteration time k
 ${\hat{S}}_{k+1}={\hat{S}}_{k}\left(F_{p}*\frac{F}{F_{k}^{\prime }}\right)$; Estimate ***S*** at the iteration time k+1
$g_{k}={\hat{S}}_{k+1}-S_{k}^{\prime }$; Calculate the gradient of ***S*** at the iteration time k
 $\alpha_{k+1}=\frac{g_{k}^{T}\cdot g_{k-1}}{g_{k-1}^{T}\cdot g_{k-1}}$; Calculate the acceleration factor at the iteration time k+1
}
**Algorithm 4:** Fast Accelerated R–L algorithm.
Initialization: ${\hat{S}}_{0}=F\;(\mathrm{or}\;{\hat{S}}_{0}={\left[1,1,\ldots ,1\right]}_{1\times N}^{T}).N\;\mathrm{is}\;\mathrm{the}\;\mathrm{number}\;\mathrm{of}\;\mathrm{elements}\;\mathrm{of}\;{\hat{S}}_{0}\;\mathrm{or}\;\mathrm{the}\;\mathrm{number}\;\mathrm{of}\;\mathrm{beams}.\;H_{p}=fft(F_{p});\;\mathrm{Calculate}\;\mathrm{the}\;\mathrm{fast}\;\mathrm{Fourier}\;\mathrm{transform}\;\mathrm{of}\;F_{p},fft\left(\ldots \right)\;\mathrm{denotes}\;\mathrm{the}\;\mathrm{fast}\;\mathrm{Fourier}\;\mathrm{transform}\;\mathrm{operation}.\;\mathrm{for}\;k=3,\;4,\;\ldots \;\{\;S_{k}^{\prime }={\hat{S}}_{k}+\alpha_{k}\left({\hat{S}}_{k}-{\hat{S}}_{k-1}\right);\mathrm{Predict}\;S\;\mathrm{at}\;\mathrm{the}\;\mathrm{iteration}\;\mathrm{time}\;k\;{\hat{H}}_{S_{k}^{\prime }}=fft\left(S_{k}^{\prime }\right);\;\mathrm{Calculate}\;\mathrm{the}\;\mathrm{Fourier}\;\mathrm{transform}\;\mathrm{of}\;S_{k}^{\prime }\;{\hat{H}}_{F_{k}^{\prime }}=H_{p}{\hat{H}}_{S_{k}^{\prime }};\;\mathrm{Calculate}\;\mathrm{the}\;\mathrm{Fourier}\;\mathrm{transform}\;\mathrm{of}\;F_{k}^{\prime }\;F_{k}^{\prime }=ifft\left({\hat{H}}_{F_{k}^{\prime }}\right);\;\mathrm{Calculate}\;\mathrm{the}\;\mathrm{inverse}\;\mathrm{Fourier}\;\mathrm{transform}\;\mathrm{of}\;{\hat{H}}_{F_{k}^{\prime }},\;ifft\left(\ldots \right)\;\mathrm{denotes}\;\mathrm{the}\;\mathrm{inverse}\;\mathrm{fast}\;\mathrm{Fourier}\;\mathrm{transform}\;\mathrm{operation}.\;\;H_{\mathrm{temp}}=fft\left(\frac{F}{F_{k}^{\prime }}\right);\;\;\mathrm{temp}=ifft\left(H_{p}^{*}H_{\mathrm{temp}}\right);H_{p}^{*}\;\mathrm{denotes}\;\mathrm{the}\;\mathrm{conjugate}\;\mathrm{of}\;H_{p}\;\;{\hat{S}}_{k+1}=S_{k}^{\prime }\mathrm{temp};\;\mathrm{Estimate}\;S\;\mathrm{at}\;\mathrm{the}\;\mathrm{iteration}\;\mathrm{time}\;k+1\;g_{k}={\hat{S}}_{k+1}-S_{k}^{\prime };\;\mathrm{Calculate}\;\mathrm{the}\;\mathrm{gradient}\;\mathrm{of}\;S\;\mathrm{at}\;\mathrm{the}\;\mathrm{iteration}\;\mathrm{time}\;k\;\;\alpha_{k+1}=\frac{g_{k}^{T}\cdot g_{k-1}}{g_{k-1}^{T}\cdot g_{k-1}};\;\mathrm{Calculate}\;\mathrm{the}\;\mathrm{acceleration}\;\mathrm{factor}\;\mathrm{at}\;\mathrm{the}\;\mathrm{iteration}\;\mathrm{time}\;k+1\;\}$

## 3. Experimental Results

### 3.1. Computation Complexity Analysis

**Figure 3:**
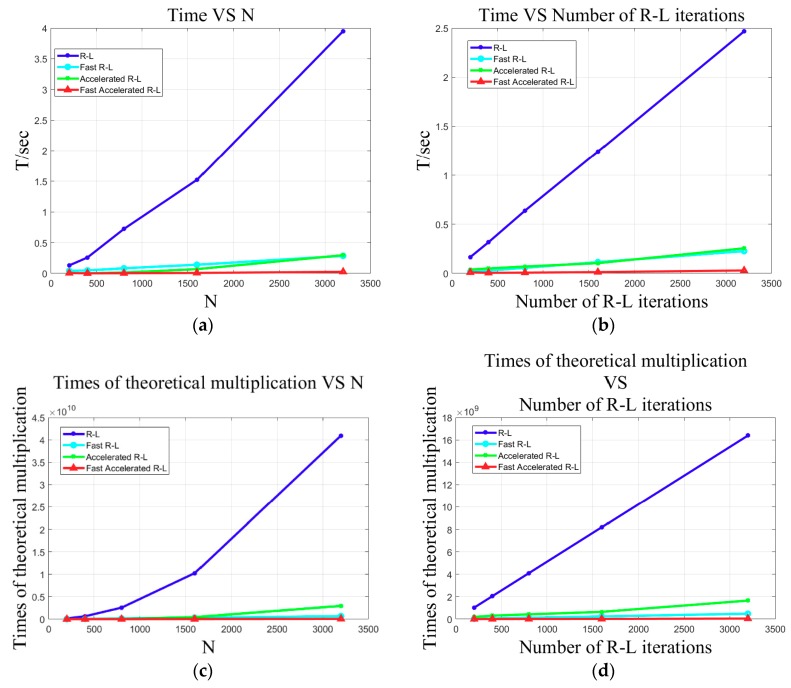
(**a**) The actual time consumption (computation complexity) and beam number N, (**b**) actual time consumption and iterative number (the higher the iterative number is, the better the results are) of the R–L algorithm, (**c**) theoretical computation complexity and beam number N, (**d**) theoretical computation complexity and iterative number of R–L algorithm.

To compare the computation complexity (here we use the length of time of computing to scale) of the four R–L algorithms, we compared and analyzed the case where the computation complexity varies as the beam number N grows when the iteration results are the same (we specify for the convenience of description that the effect of a certain number of calculation times with the Algorithm 1 iteration is the standard, and the other algorithms utilize it as the terminating condition for the iteration). Figure 3a shows that the time consumption of Algorithm 1 increased rapidly as beam number N grew. The time consumptions of Algorithm 2 and Algorithm 3 were roughly equivalent, but for Algorithm 3, it was a little less. Algorithm 4 had the least amount of calculation, which hardly increased as beam number N grew. When the beam number N equaled 3200, the time that Algorithm 1 consumed was about 150 times that of Algorithm 4, and the times that Algorithm 2 and Algorithm 3 consumed were about 12 times that of Algorithm 4. In addition, the ratio further increased as the beam number grew. Figure 3c shows a case where the theoretical amount of calculation (here we only take the time that multiplication needs into account) varies along with the growing of the beam number. This curve tendency is consistent with the experimental results.

Next, we kept the beam number N constant and analyzed a case where the amount of calculation varied along with the different iteration results (for the convenience of description, we allow the number of iterations of Algorithm 1 to represent the effect of the iteration). The higher the number of times of the iteration, the better the results were. As shown in Figure 3b, the time consumption of Algorithm 1 increased linearly as the iterative number grew. Algorithm 2 and Algorithm 3 consumed almost the same amount of time, and Algorithm 4 consumed the least amount of time. When the iterative number of Algorithm 1 was equal to 3200, the time consumption of Algorithm 1 was about 80 times that of Algorithm 4, and the time consumptions of Algorithm 2 and Algorithm 3 were about 9 times that of Algorithm 4. In addition, the ratio further increased as the beam number grew. Figure 3d shows a case where the theoretical amount of calculation varies along with the iterative number of the R–L algorithm (Algorithm 1) growing. This curve tendency is consistent with the experimental results.

Overall, we obtained the conclusion that the computation efficiency of the fast R–L algorithm (Algorithm 2) utilizing the FFT technique was greatly improved compared to the R–L algorithm (Algorithm 1). The computation efficiency of the fast-accelerated R–L algorithm (Algorithm 4) was further improved compared to the accelerated R–L algorithm (Algorithm 3). The fast-accelerated R–L algorithm (Algorithm 4) had the highest computation efficiency. Because of such a high computation efficiency, the R–L algorithm that would have required a large amount of time for calculation could be implemented in a very short time. This greatly improves the efficiency of data processing and makes the application to high-resolution multibeam sonar system in real-time possible. In the subsequent experimental data processing, we will use Algorithm 4 for the deconvolved beamforming.

The computing platform used in this experiment was an Intel® Core(TM) i5-6400 CPU with 2.7 GHz.

### 3.2. Simulation Data Experiment

In this section, the performances of deconvolved beamforming and CBF are evaluated with the simulation data. The essential parameters of the simulation were as follows: the number of array elements M = 100, array element spacing $d=\frac{\lambda }{2}$, the half of wavelength, the signal frequency was 200 kHz.

**Figure 4:**
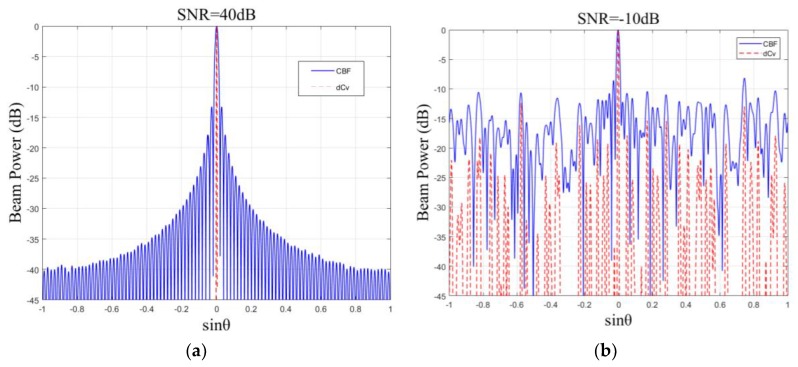
The CBF beam power (solid line) and deconvolved beam power (dashed line) for a simulated target located at a bearing of 0° with (**a**) SNR = 40 dB and (**b**) SNR = –10 dB.

Figure 4a shows the CBF beam power (solid line) and deconvolved beam power (dotted line) with the simulated data. All the beam plots were normalized with the peak set at 0 dB to show the same dynamic range. The SNR for each receiving channel was 40 dB. For CBF, we can see that the main lobe is fat and the leakage of side lobe energy is serious. For deconvolved beamforming, the main lobe was narrow and there was no side lobe leakage, which is almost an impulse function, and this is consistent with the theoretical analysis.

Next, the SNR was turned to –10 dB without changing the other parameters and the performance in a case of low SNR was studied. As shown in Figure 4b, deconvolved beamforming could still form a narrow beam at the bearing of 0° with an SNR = –10 dB and the noise power was about 4–5 dB lower than that of CBF. The explanation for this is that the noise power of CBF is composed of the equivalent noise introduced by the channel noise and the energy leakage of the main lobe, but the noise power of deconvolved beamforming is made up of only the equivalent impulse noise introduced by the channel noise. Therefore, the noise power of the reduced 4–5 dB is exactly due to the elimination of the side lobe leakage. 

**Figure 5:**
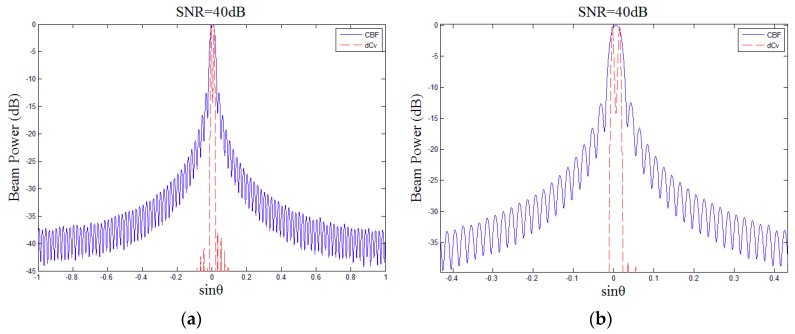
The CBF beam power (solid line) and deconvolved beam power (dashed line) for two simulated targets located at bearing of 0° and 0.7° with SNR = 40 dB, (**a**) the holistic beam power, (**b**) the local beam power of the main lobe.

Based on the case shown in Figure 4a, another same strength target was added at the bearing 0.7° to the simulation and Figure 5a was obtained. For CBF, these two targets were indistinguishable and they were regarded as one target. However, because deconvolved beamforming could easily distinguish them, this implies that the resolution of deconvolved beamforming is higher than that of CBF. Figure 5b shows the details of the main lobe.

**Figure 6:**
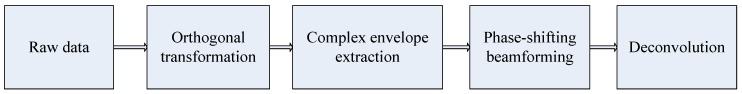
The rough procedure of the signal processing of the multibeam sonar system.

Up to now, the performance of deconvolved beamforming considering both the width of the main lobe and the side lobe level was greatly improved compared to CBF. Then, we generated the multibeam echo data that reflects a flat seabed terrain, the transmit pulse width was 0.5 ms, the receiving channel SNR was 10 dB white noise, and the rough procedure of signal processing of multibeam sonar system is shown in Figure 6.

**Figure 7:**
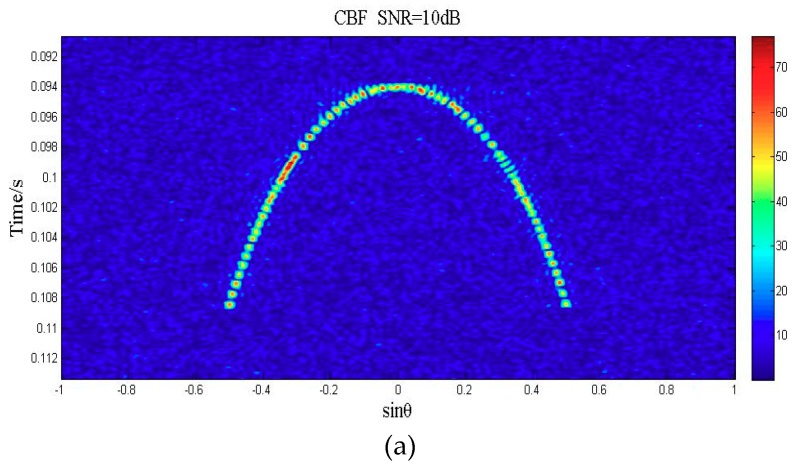

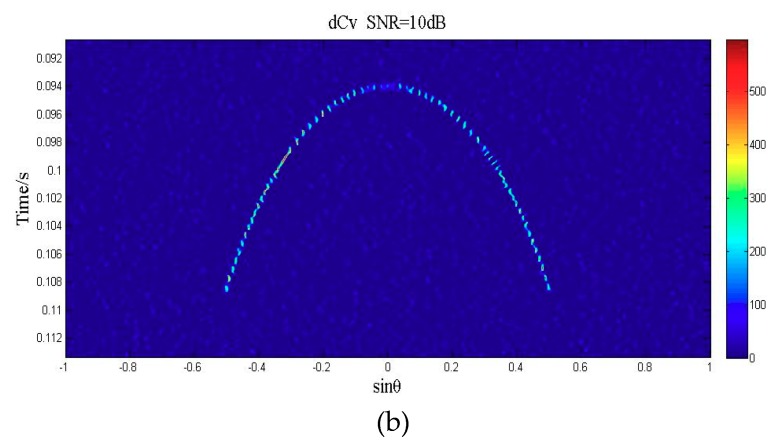
(**a**) The 2D images utilizing CBF with the simulated flat seabed echo data, (**b**) utilizing deconvolved beamforming.

Supposing the received data was compensated by TVG, the other parameters are consistent with the previous ones, and then we obtained the 2D acoustic image utilizing CBF and deconvolved beamforming. As shown in Figure 7, Figure 7a was obtained by CBF and Figure 7b was obtained by deconvolved beamforming. As shown, the image area of an echo point (that is, a target) of deconvolved beamforming is less than that of CBF, which indicates that deconvolved beamforming has a narrower main lobe than CBF. The background of the image of deconvolved beamforming is darker than CBF, which indicates that deconvolved beamforming has a lower side lobe level than CBF. 

### 3.3. Real Data Experiment

#### 3.3.1. Experimental Data Results of the Multibeam Sonar System

In this section, we utilize the data from outfield experiments to analyze the performance of CBF and deconvolved beamforming. The equipment used in the experiment was a shallow water multibeam sonar system developed by the research group where the authors work, the number of array elements was M = 100, the array element spacing was $d=\frac{\lambda }{2}$, the signal frequency was 200 kHz, CW pulse, and a pulse width 0.5ms. The experimental site was an open shallow sea area with flat topographic topography, the level of sea condition was less than four, and the hydrologic condition was good.

**Figure 8:**
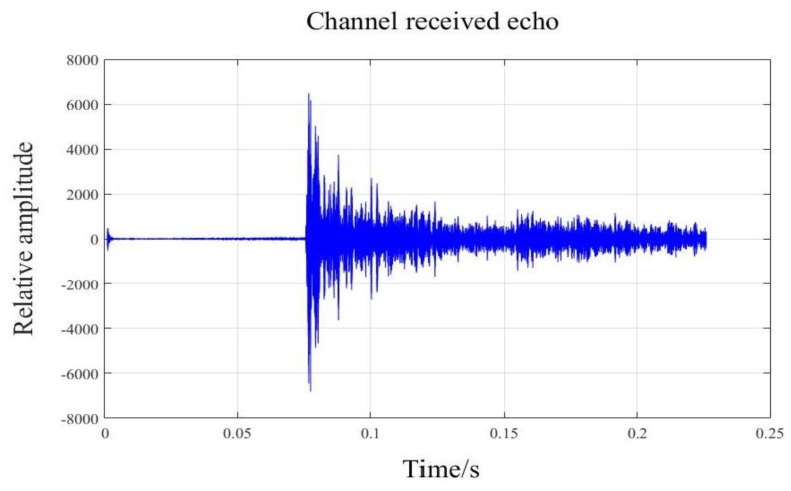
A single channel waveform in time domain.

**Figure 9:**
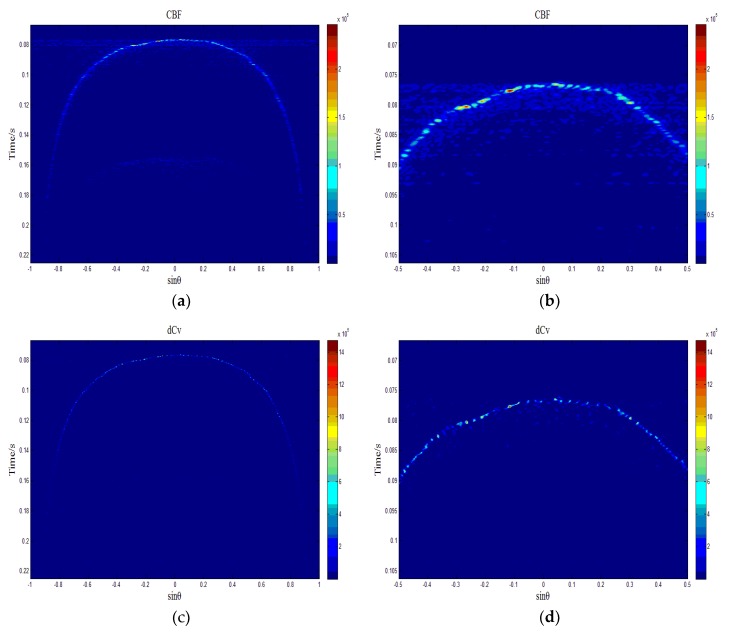

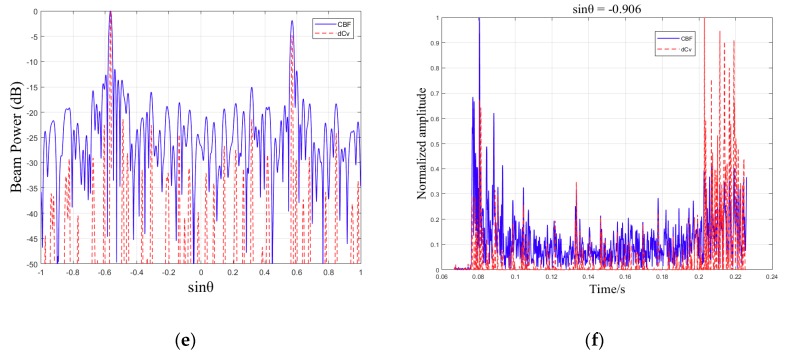
The experimental data processing results of a multibeam sounding system, (**a**) 2D acoustic image obtained utilizing CBF, (**b**) the enlarged result of the middle and upper part of (a), (**c**) 2D acoustic image obtained utilizing deconvolved beamforming, (**d**) the enlarged result of the middle and upper part of (c), (**e**) CBF beam power (solid line) and deconvolved beam power (dashed line) for t = 0.0933 s, (**f**) the echo strength (normalized) so that the sinusoidal value (sin θ) of the beam angle is −0.906 and varies along with time, and the solid line is obtained by CBF and the dashed line is obtained by deconvolved beamforming.

Figure 8 shows a single channel waveform in the time domain, which was compensated by TVG. The raw data was processed according to the procedure shown in Figure 6, and then we obtained the processed results of CBF and deconvolved beamforming, and we abstracted the beam power at the time 0.0933 s. As shown in Figure 9e, there are two main lobes, and this is because the experimental terrain was relatively flat, which led to the emergence of two strong echo points on the time slice. The main lobe width of deconvolved beamforming was narrower than that of CBF, and the noise level of deconvolved beamforming was about 5 dB lower than that of CBF, which is consistent with the simulated case above.

Figure 9a shows the 2D image obtained by utilizing CBF. It is a convex arc and it can be roughly conjectured that the actual terrain is a relatively flat terrain. The beam (that is, the vertical incident and received) located in the top of the arc has the strongest main lobe and its side lobe leakage is obvious, which is called the tunnel effect [16]. Figure 9b is the enlarged result of the middle and upper part of Figure 9a, and more details of the side lobe leakage can be seen. If the strength of the side lobe is higher than the real echo of the same beam, which usually exists in the beam of small grazing angle, the estimate of the time of arrival (TOA) is wrongly regarded as being where the side lobe is using the energy center method [17]. Figure 9f shows this case, where the sinusoidal value (sin θ) of the beam angle is −0.906, and the strength of the real echo of CBF (solid line) is obviously weaker than that of the side lobe generated by the beam of the arc’s top. There will be a mistake if the energy center method is used to estimate TOA under these circumstances. For deconvolved beamforming (dotted line), the strength of the real echo is still stronger than that of the side lobe generated by the beam of the arc’s top, and the estimate of the TOA is still correct utilizing the energy center method. Figure 9c shows the 2D image obtained utilizing CBF, and the outline is the same as Figure 9a, but it is finer and clearer. The side lobe generated by the beam of the arc’s top is significantly reduced and it is obvious that the tunnel effect is suppressed. Figure 9d is the enlarged result of the middle and upper part of Figure 9c.

#### 3.3.2. Experimental Data Results of Kongsberg M3 Multibeam Sonar

**Figure 10:**
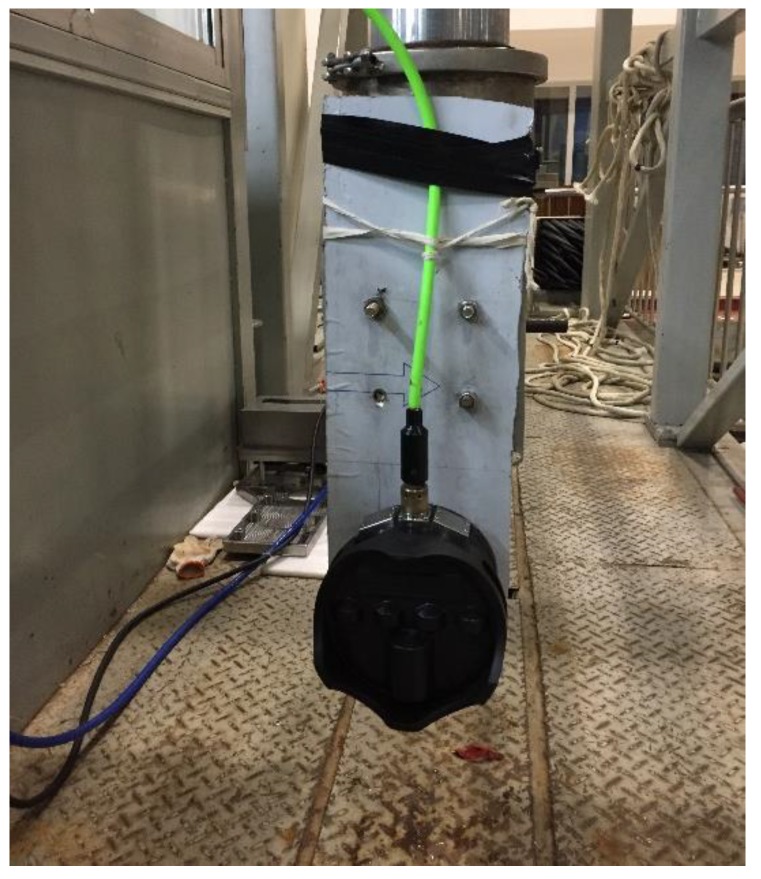
The Kongsberg M3 multibeam sonar and installation method for the experiment.

The experimental data used in this section were measured by Kongsberg M3 multibeam sonar. The frequency of the acoustic wave was 500 kHz, and the pulse width was 20 us. The experimental environment was 25 m long, 15 m wide, and 10 m deep for the full acoustic pool. The sonar and installation method used in the experiment are shown in Figure 10.

##### Two Small Ball Targets Experiment

**Figure 11:**
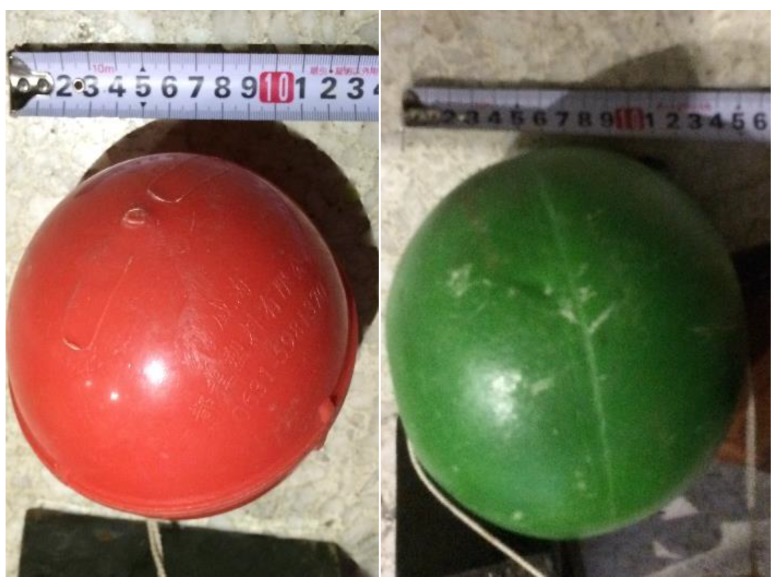
The two balls used in the experiment.

**Figure 12:**
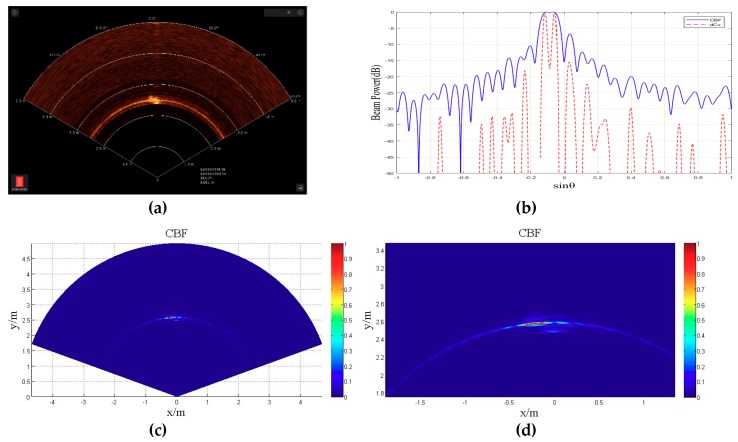

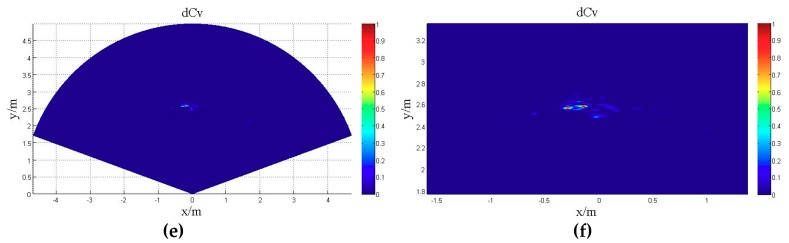
The 2D acoustic image of two close small balls by Kongsberg M3 multibeam sonar, (**a**) the display control software output, (**b**) CBF beam power (solid line) and deconvolved beam power (dashed line) at the distance where the targets exist, (**c**) 2D acoustic image obtained utilizing CBF, (**d**) the enlarged area where the two targets exist in (c), (**e**) 2D acoustic image obtained utilizing deconvolved beamforming, (**f**) the enlarged area where the two targets exist in (e).

As shown in Figure 11, as the two balls used in the experiment, the two balls in the were placed in a position 3 meters ahead of the horizontal sonar front. The geometric center was about 30 cm apart, and the 2D acoustic image is shown in Figure 11. Figure 12a is the image output from the sonar display and control software. As shown, the two ball targets show only one target in the software, and they cannot be separated. Figure 12c shows the 2D acoustic images obtained by CBF utilizing the raw data. Figure 12d is the enlarged area where the two targets exist in Figure 12c, and the two targets cannot be separated—it looks likes one target. Figure 12e shows the 2D acoustic images obtained by the deconvolved beamforming utilizing the raw data. Figure 12f is the enlarged area where the two targets exist in Figure 12e, and the two targets are completely separated. Next, we put the CBF beam power (solid line) and deconvolved beam power (dashed line) into a figure at the distance where the targets exist. To show the same dynamic range, all the beam plots were normalized with a peak set at 0 dB. As shown in Figure 12b, the CBF beam power had only a very fat main lobe, and there was only one target in the corresponding 2D acoustic image. Nevertheless, deconvolved beamforming had two narrow main lobes, and there were two targets in the corresponding 2D acoustic image. This indicates that the bearing resolution of deconvolved beamforming was better than that of CBF, and it also had a higher bearing resolution than the beamforming algorithm utilized in Kongsberg M3 multibeam sonar. It is obvious that the side lobe of the deconvolved beam power was about 7 dB lower. Accordingly, the arc bright side lobe in the 2D acoustic image of CBF vanished in the 2D acoustic image of deconvolved beamforming. This conclusion is consistent with the simulation experiment and theoretical analysis.

##### Imaging Experiment of Pool Wall

**Figure 13:**
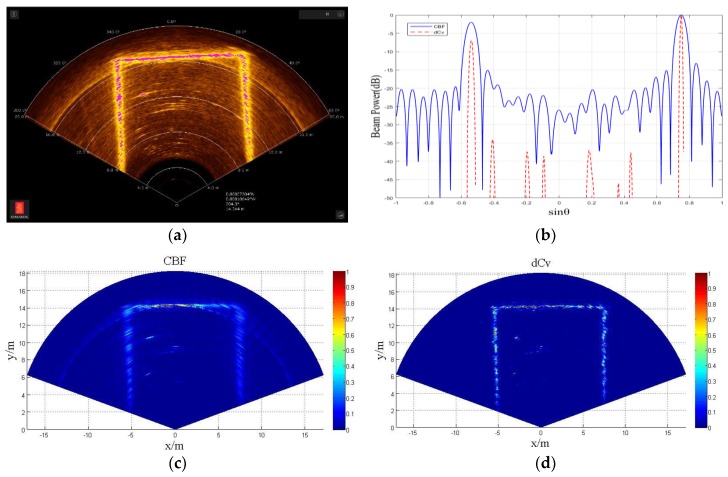
The 2D acoustic image of the pool wall by Kongsberg M3 multibeam sonar, (**a**) the display control software output, (**b**) the CBF beam power (solid line) and deconvolved beam power (dashed line) from the radial distance 10 m of the sonar, (**c**) 2D acoustic image obtained utilizing CBF, (**d**) 2D acoustic image obtained utilizing deconvolved beamforming.

As shown in Figure 13, the sonar transducer launch sector was kept horizontal, and the 2D acoustic image is obtained. Figure 13a is the 2D acoustic image output from the display control software, and there are many bright arcs formed by the side lobe leakage besides the wide bright line formed by the pool wall profile. Figure 13c is the 2D acoustic image obtained from CBF utilizing raw data. As shown, it is the same as the output image of the display control software, that is, the profile formed by the pool wall was wide, and the side lobe leakage was serious. 

Figure 13d is the 2D acoustic image obtained from deconvolved beamforming utilizing raw data, and obviously, the profile formed by the pool wall was sharper and clearer, and the bright arc caused by the side lobe leakage vanished. As shown in Figure 13b, which shows the CBF beam power (solid line) and deconvolved beam power (dashed line) from the radial distance 10 m of the sonar, the CBF beam power had two fat main lobes, and this corresponds to the wall of the two sides. The deconvolved beam power had two narrow main lobes and had about 15 dB lower of a side lobe level (the corresponding bright arc side lobe disappeared in the 2D acoustic image).

## 4. Summary and Discussion

In this paper, we proposed a new fast deconvolved beamforming algorithm to reduce the computation complexity of the R–L algorithm. Compared to signal subspace beamforming algorithms, such as MUSIC, the proposed algorithm is based on CBF, which is theoretically robust. The robustness was further demonstrated by a simulation and experimental data processing. The principle of reduction is based on the convolution theorem, which utilizes the FFT technique to calculate the convolution and correlation indirectly. Combined with the original accelerated R–L algorithm, we obtained the most efficient R–L algorithm—a fast accelerated R–L algorithm. In the application of this paper, the algorithm could reduce the computing time up to 150 times. The greater the beam number was, the higher the efficiency was. Both theoretical analysis and simulation experiments evaluated the performance of the algorithm. The algorithm made it possible for the real-time high-resolution beamforming of multibeam sonar.

At the same time, we applied the algorithm to a high-frequency horizontal array. In the application of multibeam sounding system with a frequency of 200 kHz, we obtained a 2D acoustic image of a flat seabed with a sharpening of the beam width, a suppression of the side lobe level, and a restraining the tunnel effect. In the application of Kongsberg M3 with a frequency of 500 kHz, firstly, we performed 2D acoustic imaging on two close spherical targets, and CBF could not distinguish the two targets while deconvolved beamforming could separate them. This proves that deconvolved beamforming has a higher bearing resolution than CBF. Secondly, we performed 2D acoustic imaging on the pool wall. Compared with the rough and fuzzy pool wall profile obtained by CBF, the pool wall profile obtained by deconvolved beamforming was sharper and clearer, and the 2D acoustic image of the pool wall obtained by deconvolved beamforming had no obvious bright side lobe, which proves that the deconvolved beamforming has a narrower main lobe and lower side lobe level.

Overall, the efficiency of the new fast deconvolved beamforming algorithm was higher than that of the existing deconvolved beamforming algorithm. The advantages of the deconvolved beamforming algorithm were verified in the high-frequency linear array.

## Figures and Tables

**Table 1:** The amount of calculation of four algorithms.

Algorithm	Multiplicative Times per Iteration
1	$2N^{2}+N$
2	$8N\log_{2}N+9N$
3	$2N^{2}+4N$
4	$8N\log_{2}N+12N$

**Table 2:** The differences of the four algorithms.

Algorithm	Fast	Accelerated	Computation Complexity
1	NO	NO	Most
2	YES	NO	More
3	NO	YES	Less
4	YES	YES	Least
